# Association of Herpes Zoster and Type 1 Diabetes Mellitus

**DOI:** 10.1371/journal.pone.0155175

**Published:** 2016-05-12

**Authors:** Hsin-Hung Chen, I-Ching Lin, Hsuan-Ju Chen, Su-Yin Yeh, Chia-Hung Kao

**Affiliations:** 1 School of Medicine and Public Health, Chung Shan Medical University, Taichung, Taiwan; 2 Division of Metabolism & Endocrinology, Changhua Christian Hospital, Changhua, Taiwan; 3 Nantou Christian Hospital, Nantou, Taiwan; 4 School of Medicine, Chung Shan Medical University, Taichung, Taiwan; 5 Department of Family Medicine, Changhua Christian Hospital, Changhua, Taiwan; 6 Management Office for Health Data, China Medical University Hospital, Taichung, Taiwan; 7 College of Medicine, China Medical University, Taichung, Taiwan; 8 Asia University, Taichung, Taiwan; 9 Graduate Institute of Clinical Medical Science and School of Medicine, College of Medicine, China Medical University, Taichung, Taiwan; 10 Department of Nuclear Medicine and PET Center, China Medical University Hospital, Taichung, Taiwan; University Medicine Greifswald, GERMANY

## Abstract

**Objective:**

The purpose of our study was to determine the association of type 1 diabetes mellitus (T1DM) and the risk of herpes zoster (HZ).

**Methods:**

In this cohort study, we selected 4736 patients with T1DM registered in the Catastrophic Illness Patient Database who received insulin therapy before 2003 and 18944 participants without DM who were selected by frequency matched based on sex and age. Cox proportional hazard regression analysis was used to measure the hazard ratios (HRs) of HZ in the T1DM group compared with that in the non-T1DM group.

**Results:**

Cox proportional hazard regression analysis showed that the adjusted HR of HZ was 2.38 times higher for patients in the T1DM group (95% CI = 1.77–3.19) than for those in the non-T1DM group. According to diabetes severity, mild and serious T1DM patients were associated with a higher risk of HZ (adjusted HR = 2.26, 95% CI = 1.67–3.05; and adjusted HR = 5.08, 95% CI = 2.66–9.71, respectively) than subjects without T1DM.

**Conclusion:**

Patients with T1DM are at a higher risk of HZ than those without T1DM.

## Introduction

Patients with diabetes or immunocompromised conditions are at a high risk of herpes zoster (HZ) [1,2].Impaired cell-mediated immunity was observed in patients with type 1 diabetes (T1DM), making them susceptible to HZ infections [3]. Latent varicella herpes virus can cause HZ in patients with impaired cell-mediated immunity. In Taiwan, diabetes is one of the 10 leading causes of death [4], and the incidence of HZ is 4.89 patients per 1000 person-years among all age groups; diabetes is an independent risk factor for HZ in Taiwan [1].The incidence of HZ in Caucasians is1.2–4.8 per 1000person-years [5–7], lower than that in Asians. T1DM and not T2DM were associated with HZ infections in a study [8], but no possible explanations were provided. In this study, we evaluated the association between T1DM and HZ.

## Methods

### Data Source

The National Health Insurance (NHI) program is a universal insurance program in Taiwan established in 1995; it was reformed from 13 insurance-related systems and covers nearly 99% of the approximately 23 million people in Taiwan. All claims data are linked to patient information and are anonymized and maintained by the NHI reimbursement database. Each patient is assigned a unique identification number, and the scrambled data is released for public research. In this study, we used data sets from the Registry for Longitudinal Health Insurance Database (LHID2000) and Catastrophic Illness Patient Database (CIPD). The demographic characteristics of the patient (including sex and age) and all clinical visit records (including prescription details and diagnoses coded according to the International Classification of Diseases, Ninth Revision, Clinical Modification [ICD-9-CM]codes) are available in these data files. Each data set can be interlinked using the unique personal identification number.

### Study Population

In this cohort study, we selected 5167 prevalent patients with T1DM (ICD-9-CM 250, excluding type 2 diabetes mellitus) registered in the CIPD who received insulin therapy before 2003; this group was defined as the T1DM group. In Taiwan, T1DM is one of the 30 major disorders categorized as a catastrophic illness under the NHI program. In our study, January 1, 2003, was the index date. We further excluded patients with missing age and sex information at baseline (n = 309) and/or with the history of HZ (ICD-9-CM 053) (n = 122) from the T1DM group. For each patient with T1DM, four persons without a history of DM (ICD-9-CM 250) and HZ were randomly selected from the LHID2000 and frequency matched by sex and age; this group was defined as the non-T1DM group. The T1DM and non-T1DM groups were followed until end date (i.e. the date of HZ diagnosis, withdrawal from the NHI program, death, or December 31, 2011, whichever occurred first).

To evaluate an individual's severity of diabetes complication, we applied adapted Diabetes Complication Severity Index (aDCSI), which does not include laboratory test results as an indicator of diabetes severity [9,10]. The aDCSI consists of severity score (0, 1, and 2) from 7 categories of diabetic complications: retinopathy, nephropathy, neuropathy, cerebrovascular, cardiovascular, peripheral vascular disease, and metabolic disease, and ranges from 0 to 13. The progression of diabetes was defined as increased aDCSI score each year (the average change of aDCSI from the index date to the end date).

The demographic characteristics were sex (men and women) and age (< 20, 20–39, and ≥ 40 y). Cancer (ICD-9-CM 140–208), depression (ICD-9-CM 296.2, 296.3, 300.4, and 311), heart failure (ICD-9-CM 428), renal disease (ICD-9-CM 580–589), systemic lupus erythematous (ICD-9-CM710.0), and rheumatoid arthritis (ICD-9-CM714) before the index date were identified as comorbidities. Medications that could affect HZ progression, such as statins, angiotensin receptor blockers (ARBs), and angiotensin converting enzyme inhibitors (ACEIs), and prednisolone, were included as analysis variables. Only medications prescribed before the end date were considered.

### Ethics Statement

The NHIRD encrypts patient personal information to protect privacy and provides researchers with anonymous identification numbers associated with relevant claims information, including sex, date of birth, medical services received, and prescriptions. Therefore, patient consent is not required to access the NHIRD. This study was approved to fulfill the condition for exemption by the Institutional Review Board (IRB) of China Medical University (CMUH104-REC2-115). The IRB also specifically waived the consent requirement.

### Statistical methods

Differences in demographic characteristics, comorbidities, and medications between the T1DM and non-T1DM groups were analyzed using the chi-square test for categorical variables and the Student *t* test for continuous variables. Person-years of follow up were calculated for each patient from the index date to the date of HZ diagnosis, loss to follow-up, death, or end of follow-up (December 31, 2011). The incidence density rate (per 1000 person-years) was assessed for each subgroup. Survival was analyzed using the Kaplan–Meier method, and the log-rank test was used to compare survival distributions between the groups. After adjusting for sex, age, comorbidities, and medications, we performed a Cox proportional hazards analysis. Stratified analysis was performed to examine the association between T1DM and the risk of HZ, in which the patients were stratified by sex, age, and the presence of comorbidities. Furthermore, we evaluated the association between diabetes severity and risk of HZ. Hazard ratios (HRs) and 95% confidence intervals (CIs) were calculated to quantify the risk of HZ.

All data processing and statistical analyses were performed using SAS 9.3 (SAS System for Windows, SAS Institute, Cary, NC, USA). R software (R Foundation for Statistical Computing, Vienna, Austria) was used for plotting the Kaplan–Meier curves. The significance level was defined at alpha = 0.05. Thus, two sided p < 0.05 was considered statistically significant.

## Results

In this retrospective cohort study, 23 680 participants—4736 patients with T1DM and 18 944 participants without T1DM—were analyzed. Their demographic characteristics, comorbidities, and medications are reported in Table 1. The T1DM group had more women than men (53.48% vs 46.52%), and the average age of the patients was 26.72 years (standard deviation = 14.25 y). Patients in the T1DM group were more likely to exhibit comorbidities, such as cancer, depression, heart failure, and renal disease, and use medication, such as statins, ARBs, ACEIs, but not prednisolone.

**Table 1:** Baseline demographic factors and comorbidity of study participants according to T1DM status.

	Non-T1DM group N = 18944		T1DM group N = 4736		p-value
Variable	n	%	n	%	
**Sex**					0.99
Women	10132	53.48	2533	53.48	
Men	8812	46.52	2203	46.52	
**Age, years**					0.99
< 20	6956	36.72	1739	36.72	
20–39	8716	46.01	2179	46.01	
≥ 40	3272	17.27	818	17.27	
Mean (SD) ^†^	26.73	(14.32)	26.72	(14.25)	0.95
**Comorbidity**					
Depression	249	1.31	163	3.44	<0.001
HF	45	0.24	46	0.97	<0.001
Renal disease	263	1.39	662	13.98	<0.001
Cancer	69	0.36	55	1.16	<0.001
SLE^‡^	14	0.07	2	0.04	0.75
RA^‡^	4	0.02	2	0.04	0.34
**Drug**					
Statin	835	4.41	1823	38.49	<0.001
ARB	805	4.25	1395	29.46	<0.001
ACEI	1079	5.70	1697	35.83	<0.001
Prednisolone	12579	66.40	2906	61.36	<0.001

Abbreviation: T1DM, type 1 diabetes mellitus; SD, standard deviation; HF, heart failure; SLE, systemic lupus erythematous; RA, rheumatoid arthritis;ARB, angiotensin receptor blocker; ACEI, angiotensin converting enzyme inhibitor.

^†^Student’s t-test.

^‡^ Fisher’s exact test.

**Fig 1:**
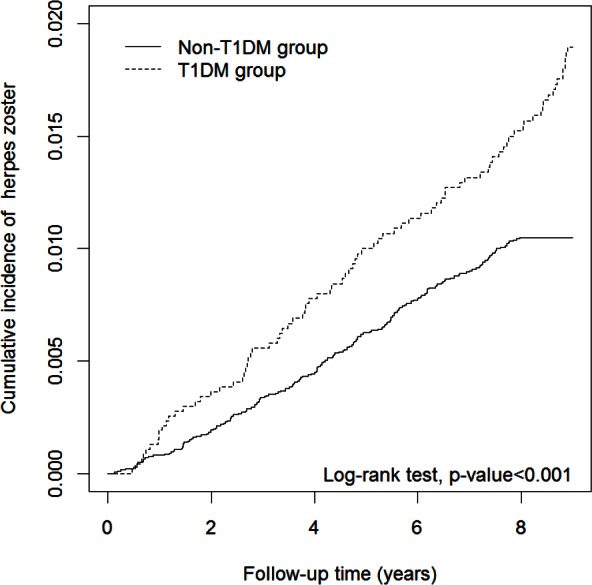
Cumulative incidence curves of herpes zoster for groups with and without T1DM. T1DM, type 1 diabetes mellitus.

The results of the log-rank test and the cumulative incidences of HZ are shown in Fig 1. The Kaplan–Meier analysis was used to reveal the risk of HZ during follow-up in both groups. The cumulative incidence of HZ was significantly higher in the T1DM group than in the non-T1DM group (p<0.001).

During the follow-up period, 85 and 195 patients in the T1DM and non-T1DM groups, respectively, were diagnosed with HZ, at an incidence density rate of 2.11 and 1.19 per 1000 person-years. Cox regression analysis showed that the adjusted HR of HZ was 2.38 times higher for the T1DM group (95% CI = 1.77–3.19) than for the non-T1DM group. Sex-specific analysis indicated that the incidence density rates of HZ in women and men in the T1DM group were 2.03 and 2.21 per 1000 person-years, respectively. These incidence density rates in the T1BM group were higher than those in the non-T1DM group (1.37 and 0.99 per 1000 person-years for women and men, respectively). The risk of HZ was significantly higher in the T1DM group than in the non-T1DM group (for both women and men), at an adjusted HR of 1.96 (95% CI = 1.31–2.92) and 3.03 (95% CI = 1.95–4.71), respectively. Age-specific analysis indicated that the incidence density rate of HZ increased with age in both the groups. In all age groups, patients with T1DM had a significantly higher risk of HZ than those without T1DM. The adjusted HR of HZ was 1.93 (95% CI = 1.01–3.69) in patients aged 20 years or younger, 2.49(95% CI = 1.49–4.17) in patients aged 20–39 years, and 1.93 (95% CI = 1.23–3.02) in patients older than 40 years. In participants without comorbidity, the risk of HZ was 2.59 times higher (95% CI = 1.89–3.55) in the T1DM group than that in the non-T1DM group (Table 2).

**Table 2:** Incidence density rates and hazard ratios of herpes zoster according to T1DM status stratified by sex, age, and comorbidity.

	T1DM						Compared to non-T1DM group			
	No			Yes			HR (95% CI)			
Variable	Event no.	Person-years	IR	Event no.	Person-years	IR	Crude	p-value	Adjusted ^‡^	p-value
**Overall**	195	163212	1.19	85	40226	2.11	1.78 (1.38–2.29)	<0.001	2.38 (1.77–3.19)	<0.001
**Sex**										
Women	120	87745	1.37	44	21703	2.03	1.49 (1.06–2.11)	0.02	1.96 (1.31–2.92)	0.001
Men	75	75467	0.99	41	18524	2.21	2.24 (1.53–3.28)	<0.001	3.03 (1.95–4.71)	<0.001
**Age, years**										
< 20	34	60724	0.56	20	15368	1.30	2.35 (1.35–4.08)	0.002	1.93 (1.01–3.69)	0.04
20–39	65	75012	0.87	31	18616	1.67	1.94 (1.26–2.97)	0.002	2.49 (1.49–4.17)	<0.001
≥ 40	96	27476	3.49	34	6242	5.45	1.57 (1.06–2.32)	0.02	1.93 (1.23–3.02)	0.004
**Comorbidity status** ^**†**^										
No	177	158116	1.12	68	33872	2.01	1.80 (1.36–2.38)	<0.001	2.59 (1.89–3.55)	<0.001
Yes	18	5097	3.53	17	6354	2.68	0.77 (0.40–1.49)	0.42	1.24 (0.54–2.81)	0.61

Abbreviation: T1DM, type 1 diabetes mellitus; IR, incidence density rate, per 1,000 person-years; HR, hazard ratio; CI, confidence interval.

^†^ Patients with any one of depression, heart failure, renal disease, cancer, systemic lupus erythematous, and rheumatoid arthritis were classified as the comorbidity group.

^‡^ Model mutually adjusting for sex, age (continuous), depression, heart failure, renal disease, cancer, systemic lupus erythematous, rheumatoid arthritis,statin, angiotensin receptor blocker, angiotensin converting enzyme inhibitor, and prednisolone.

The results of an additional analysis in which the T1DM group was stratified by different severity are listed in Table 3. We found that the risk of HZ increased, from 2.26 (95% CI = 1.67–3.05) for mild T1DM patients (aDCSI Score = 0–0.5 per year) to 5.08 (95% CI = 2.66–9.71) for serious T1DM patients (aDCSI Score > 0.5 per year) than non-T1DM patients.

**Table 3:** Incidence density rates and hazard ratios of herpes zoster in different groups.

Change in aDCSI Score per year	N	Event no.	IR	HR (95% CI)			
				Crude	p-value	Adjusted ^‡^	p-value
**Non-T1DM group**	18944	195	1.19	1.00		1.00	
**T1DM group**							
0–0.5	4379	73	1.93	1.62 (1.24–2.12)	<0.001	2.26 (1.67–3.05)	<0.001
> 0.5	357	12	5.06	4.29 (2.39–7.68)	<0.001	5.08 (2.66–9.71)	<0.001
p for trend				<0.001		<0.001	

Abbreviation: aDCSI, adapted Diabetes Complication Severity Index; T1DM, type 1 diabetes mellitus; IR, incidence density rate, per 1,000 person-years; HR, hazard ratio; CI, confidence interval.

^‡^ Model adjusting for sex, age (continuous), depression, heart failure, renal disease, cancer, systemic lupus erythematous, rheumatoid arthritis, statin, angiotensin receptor blocker, angiotensin converting enzyme inhibitor, and prednisolone.

## Discussion

### Diabetes and the Risk of HZ

In general, patients with diabetes are more prone to infections than those without diabetes [11,12].In Taiwan, the incidence of HZ was 4.89 cases per 1000 person-years among all age groups, and 20.60% of patients with HZ had diabetes, with multivariate analysis confirming diabetes as an independent risk factor for HZ [1].In our study, the incidence density rate of HZ was 2.11 per 1000 person-years in the T1DM group, and Cox regression analysis showed that the adjusted HR of HZ was 2.38 times higher for patients with T1DM than for those without T1DM.Women and men in the T1DM group had a significantly higher risk of HZ than those in the non-T1DM group, with an adjusted HR of 1.96 and 3.03, respectively. Cell-mediated immunity is critical for preventing and controlling varicella-zoster virus (VZV) infection. One small study mentioned that patients with diabetes might have low cell-mediated immunity to VZV [13]. Therefore, diabetes coupled with impaired cell-mediated immunity was a possible mechanism for the high HZ risk [2].Our finding is consistent with that of other studies on diabetes and HZ [14].Diabetes is not only a factor for HZ infection but also for postherpetic neuralgia [15,16]. In a Taiwanese study, the hospitalization rate for HZ was 16.1 cases per 100 000 person-years, and the cost for individual home care and hospitalized cases were approximately €53.30 and €1224.70, respectively [1].In a previous study[8], the adjusted odds ratio for HZ in patients with T1DMwas lower than our study(1.27 vs 2.38), possibly because of racial [17] or genetic differences; these results imply that HZ is an emerging public health problem for Asian patients with T1DM.

### Medications and HZ in T1DM

A previous study reported that statin use increases the risk of HZ in Asians [18]. Thus, in our study, statin use was an adjusted factor. ARBs and ACEIs were widely prescribed for hypertension or diabetic nephropathy. We also added these medications in our analysis. Steroid was also a possibility to alter immunity. In our study, steroid prescription was lower in T1DM group, so we could exclude the associations of steroid between T1DM group and HZ. Poor glycemic control possibly enhances impaired cell-mediated immunity. In the real world, compliance to insulin use, lifestyle modification and diet control are possible reasons to decide the quality of diabetic control. It was difficult to collect the detail data from our database such as lifestyle, diet, compliance to insulin or insulin dose and laboratory data. To evaluate an individual's severity of diabetes complication, we applied adapted Diabetes Complication Severity Index (aDCSI), which does not include laboratory test results as an indicator of diabetes severity. The aDCSI consists of severity score (0, 1, and 2) from 7 categories of diabetic complications. We found that the risk of HZ increased, from 2.26 (95% CI = 1.67–3.05) for mild T1DM patients (aDCSI Score = 0–0.5 per year) to 5.08 (95% CI = 2.66–9.71) for serious T1DM patients (aDCSI Score > 0.5 per year) than non-T1DM patients.

### T1DM and HZ

Certain HLA antigens were reported to have a positive association with symptomatic herpes disease [19]. Another study reported HLA as a susceptible T1DM gene in Asia [20]. Thus, HLA could influence HZ infections. Additional studies that explore associations among T1DM and HZ are warranted.

A United Kingdom-based study reported that only T1DMand not T2DM exhibited some association with HZ [8]. A recent United States-based reported a lack of evidence for the risk of HZ in patients with T1DM [21].Our study showed that Asians with T1DM may be at a risk of HZ.

### Conclusions

Several studies have reported DM as an independent risk factor for HZ. However, according to most studies, only patients with T2DM were at a risk of HZ. Compared with Caucasians, Asians have a lower incidence of T1DM (0.4±1.1 cases/year/100 000 individuals) [20]; however, Taiwanese with T1DM have a higher risk of HZ.

### Limitations

First, the data on potential confounding factors, such as nutritional status, smoking, and alcohol consumption, are lacking in the LHID2000 and CIPD databases. Second, the quality of diabetes control may be a relevant determinant of HZ risk, but this data was lacking in our databases. Third, because of the low incidence of T1DM in Asia, adjusting for the risk of HZ for medications such as immuno-suppressants and conditions, such as previous bone marrow or organ transplantation, could not be performed; the adjustment was performed only for the comorbidities and basic demographic characteristics in our meta-analysis. Fourth, underestimated prevalence of HZ in T1DM group is true because of ICD-9 limitation. In the other hand, if we could collect all of these T1DM patients with HZ, we believe that the associations between T1DM and HZ will be stronger.

## Supporting Information

S1 STROBE Checklist STROBE checklist. (DOC)
